# Three cases of collared owlet depredation on the green‐backed tit within nest boxes

**DOI:** 10.1002/ece3.11083

**Published:** 2024-03-04

**Authors:** Nehafta Bibi, Qingmiao Yuan, Caiping Chen, Shaolian Chen, Yubao Duan, Xu Luo

**Affiliations:** ^1^ Key Laboratory for Forest Resources Conservation and Utilization in the Southwest Mountains of China, Ministry of Education/Faculty of Biodiversity and Conservation Southwest Forestry University Kunming China

**Keywords:** collared owlet, green‐backed tit, hole size, nest predation

## Abstract

The main cause of the reproductive failure of cavity‐nesting birds is nest predation, even though cavity nests protect from numerous predators. To study the breeding biology of the green‐backed tit (*Parus monticolus*) and to promote the reproduction of some other avian cavity breeders, we placed 245 nest boxes in the Zixi Mountain, southwest China. We collected breeding data by regularly checking the artificial nest boxes, three cases of green‐backed tits being predated by collared owlet (*Glaucidium brodiei*) were confirmed by the video recordings. Larger mammals, chipmunks, squirrels, sparrowhawks, jays, and snakes have been identified as common predators of cavity‐nesting birds in high‐latitude regions of the northern hemisphere. Limited research in tropical and subtropical regions, particularly in Asia, has demonstrated squirrels and snakes as common predators of cavity‐nesting birds. A gap in avian predators to cavity‐nesting birds exists in the current knowledge. Hence the three cases of collared owlet's depredation reveal a new danger to green‐backed tits, broadening our knowledge of the dynamics of cavity‐nesting birds. In all three cases, the artificial box's entrance hole was only 5 × 5 cm in size and has not been expanded or poked. These findings provided evidence that the collared owlet is the predator of nestlings and adult green‐backed tit breeding in artificial boxes, which emphasized a reevaluation of predator–prey interactions. Therefore, for effective breeding of the green‐backed tit, we suggest to choose a hole size of 3 × 3 cm that is appropriate for its body size.

## Graphical abstract

To study the breeding biology of the green‐backed tit (*Parus monticolus*) and to promote the reproduction of some other avian cavity‐breeders, we placed 245 nest boxes in the Zixi Mountain in central Yunnan, China. Three cases of green‐backed tits being predated by collared owlet (*Glaucidium brodiei*) were confirmed by the video recordings. In all three cases, the artificial box's entrance hole was only 5 × 5 cm in size and has not been expanded or poked. These findings provided evidence that the collared owlet is the predator of nestlings and adult green‐backed tit breeding in artificial boxes, which emphasized a reevaluation of predator–prey interactions. Therefore, for effective breeding of the green‐backed tit, we suggest to choose a hole size of 3 × 3 cm that is appropriate for its body size.

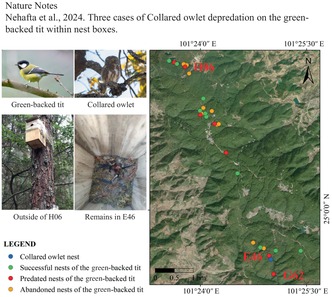

## 1 INTRODUCTION

Hole size is an important aspect of the suitability of a nest site for birds that breed in cavities (Le Roux et al., 2016). Smaller holes can offer more security against competition and predators than larger entrances which are less desirable to small birds (Juškaitis, 2021; Valera et al., 2019). According to previous studies, smaller holes were preferred because they can keep numerous predators from going through and getting to the nest's contents (Walankiewicz, 1991). In addition to having a sufficient hole size, a nest box's depth is also thought to have an impact on how many birds breed there because shallow nests are more vulnerable to predation (Nilsson, 1984). A secure cavity should have a hole that is proportional to the occupant's body size and should be deep enough to prevent the avian occupant from being easily damaged by predators (Le Roux et al., 2016; Wesołowski, 2002). Hence, smaller, more appropriate entrance holes and sufficient depth provide suitable nest sites for cavity‐nesting birds, enhancing their safety against potential predators (Yu et al., 2021).

To better understand ecological interactions and predict the frequency of nest loss with the aim of conservation, it is helpful to understand the identity of predators and their modes of predation (Chalfoun et al., 2002; Lima, 2002; Schmidt, 1999). In addition, it aids in our comprehension of the selective factors that influence parental and offspring antipredator strategies (Ibáñez‐Álamo et al., 2015). It is difficult to observe the predation process firsthand, though, due to the regularity and secrecy of natural predation events. As a result, researchers utilize the remnants of abandoned nests to make irrational inferences about potential predators (Williams & Wood, 2002). However, actual nest predators may differ from these assumptions (Peterson et al., 2004). Video cameras are widely used to monitor the behavior of breeding birds and sometimes to record their predation. For instance, it was reported based on video evidence that a single Eurasian jay (*Garrulus glandarius*) attacked a female Japanese tit (*Parus minor*) who was incubating nine eggs at a nest box because a woodpecker had significantly widened the entrance (Yin et al., 2023). In another case, the video recording showed the process of multiple host individuals of the Oriental reed warbler (*Acrocephalus orientalis*) mobbing and attacking a female common cuckoo (*Cuculus canorus*) in the field (Zhao et al., 2022). Through video monitoring, accumulated evidence of natural predation provided an understanding and identification of predator's actions, which is crucial for improving conservation efforts by forecasting nest loss frequencies (Thompson III & Ribic, 2012).

Predators such as squirrels (*Tamiasciuris* spp.), chipmunks (*Tamias* spp.), long‐tailed weasel (*Mustela frenata*), and forest mice (*Peromyscu*s spp.) are the most common ones associated with cavity nest predation in the western United States and Canada (Fontaine & Martin, 2006; Li & Martin, 1991; Walters & Miller, 2001). While in central Texas, Sperry et al. (2008) reported snakes as predators of cavity‐nesting birds. In northwest England, vole (*Myodes glareolus*), weasel (*M. nivalis*), red squirrel (*Sciurus vulgaris*), and pine marten (*Martes martes*) were identified as predators that prey on cavity‐nesting birds (Wesol̸owski, 2017). Predators, including Japanese marten (*Martes melampus*), Japanese rat snakes (*Elaphe climacophora*), and Jungle crows (*Corvis macrorhynchos*), were reported to pray on passerines in Japan (Suzuki & Ueda, 2013). The red squirrel, the common chipmunk, and the Eurasian sparrowhawk (*Accipiter nisus*) were all identified as nest predators of cavity‐nesting passerines in Zuojia Nature Reserve of northeast China (Shen et al., 2023; Yu et al., 2020). However, within the same nature reserve, Yin et al. (2023) found one Eurasian jay attacking an adult incubating Japanese tit. Hence, larger mammals, chipmunks, sparrowhawks, jays, and snakes have been found common predators of cavity‐nesting birds in high‐latitude regions ranging from England to North America, Canada, North China, and Japan. Few studies in tropical and subtropical areas, particularly in Asia, have revealed that squirrels and snakes are common predators of cavity‐nesting birds (e.g., Mo et al., 2023; Quan & Li, 2015). Interestingly, studies are somewhat limited in these places than in high‐latitude regions, and prior studies frequently highlight mammals and reptiles as predators of cavity‐nesting birds (Cai et al., 2018; Liu et al., 2023). A gap in avian predators to cavity‐nesting birds exists in the current knowledge. In northern America including the southeast United States, great spotted woodpecker (*Dendrocopos major*) and Northern pygmy owls (*Glaucidium gnoma*) were known to prey on small singing birds but Eurasian pygmy owls (*G. passerinum*) were found attacking them occasionally (Holt & Leroux, 1996; McPherson & Brown, 1981). However, whether these owl species prey on eggs or nestlings of cavity‐nesting birds was not mentioned in these studies. Here we reported several cases of nest predation of green‐backed tit (*Parus monticolus*), an East Asian montane cavity‐nesting passerine species (BirdLife International, 2016), which was predated by an owlet (*G. brodiei*) from the subtropical area of southwest China.

## 2 METHODS

**FIGURE 1:**
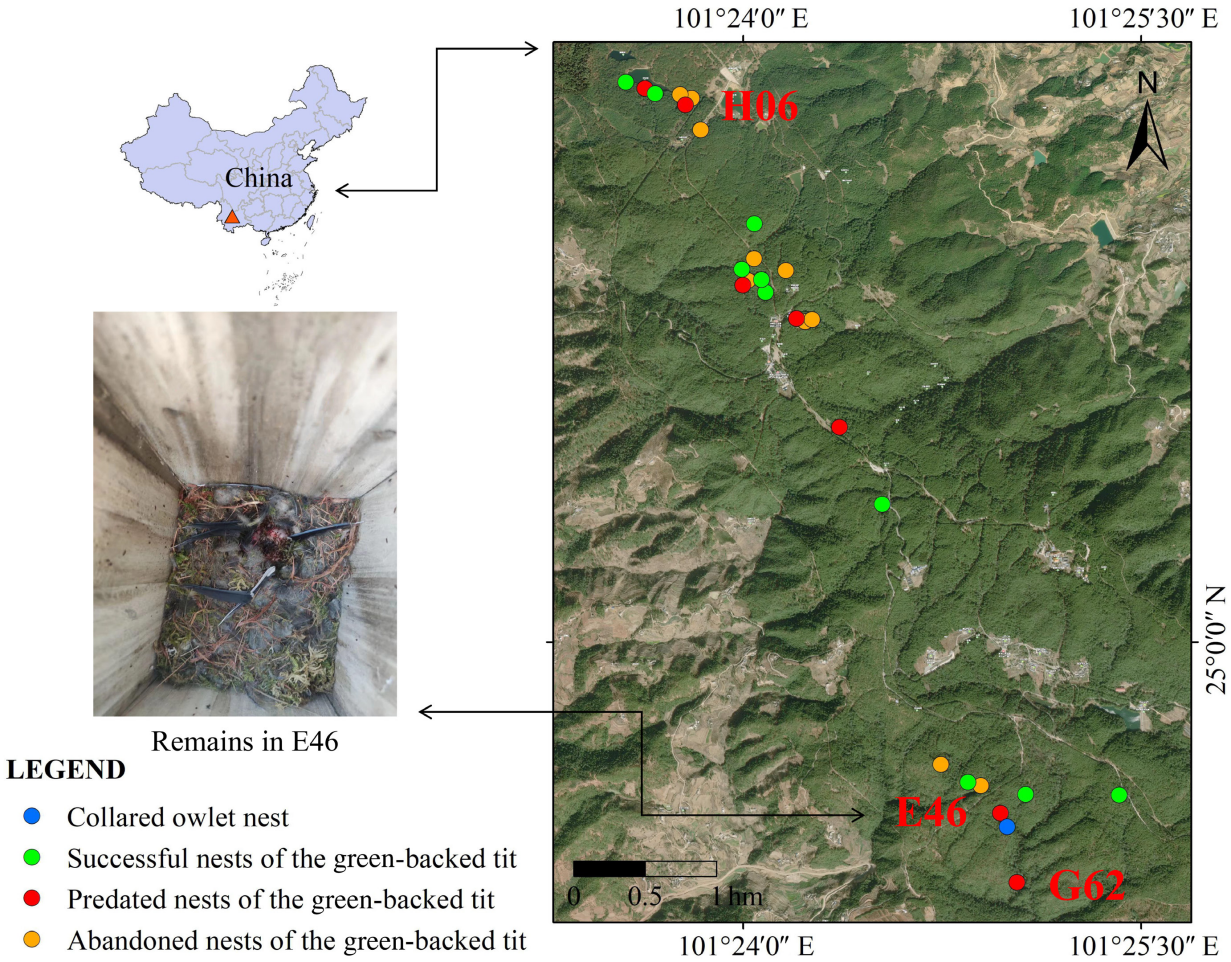
Zixi Mountain Provincial Nature Reserve in Yunnan Province, southwest China and prey remains of green‐backed tit's nestlings.

From 2019 to 2023, we installed 245 artificial nest boxes inside the Zixi Mountain Provincial Nature Reserve in Yunnan Province southwest China (101°22′49″–101°26′07″ E, 24°58′58″–25°04′01″ N, 1950–2502 m above sea level; Figure 1). The nest boxes were placed firstly to study the green‐backed tit's breeding ecology (to be published) and to promote some other avian cavity breeder's reproduction, for example, nuthatch, treecreeper, and woodpecker (as mentioned by Mo et al., 2023). This nature reserve has a total area of 160 km^2^, predominantly covered by coniferous and mixed coniferous forests (Mo et al., 2023). The reserve's forest area is 151.211 km^2^ and its nonforested area is 24.92 km^2^. The forest mostly contains *Castanopsis orthacantha* woods, Yunnan pine (*Pinus yunnanensis*), and Huashan pine (*P. armandii*) (Mo et al., 2023). The nest boxes were hung on trees between 2.5 and 4 m high and were made of pine wood, 25 × 14 × 12 cm (height × length × width) in size, with an entrance hole of 5 cm in diameter. The linear distance between each nest box was more than 50 m (Zhang et al., 2021).

Throughout the green‐backed tit breeding season, which in our research area lasts from April to June, the nest boxes were checked every second day. When we found eggs, the laying date was determined by counting backward from the number of eggs, since tits typically lay one egg each day (Perrins, 1970). We considered the next box as active if it contained eggs or chicks, or had an incubating female (Hanzelka et al., 2023). When a nest box was confirmed to be occupied for breeding, we monitored the box from 6:00 a.m. to 08:00 p.m. using digital cameras (HD99S‐10, LooSafe, Dongguan, Guangzhou, China). The lens of the camera was placed into the box from the top cover and the cameras were wire connected with extra battery outside. We made continuous recordings until the nestling fledged or was predated. In total, we monitored 27 active nest boxes of green‐backed tit during the breeding season in 2023. From the 27 nests, we were able to collect 2485 h of video recordings, of which, seven nests were attacked by predators, 10 were abandoned, and another 10 were successful. Every video recording was identified by its nest box ID. Then, we watched the videos to witness the occurrences of nest predation. Our team of four researchers completed the fieldwork and video checking.

## 3 RESULTS

We found three cases of green‐backed tit being predated by collared owlet, each case occurred in one artificial box (box ID: E46\G62\H06). When we detected the predation from the video recorded within the tits' nesting box, we also checked the videos from the owlet's box of the same day as one artificial nest box was occupied by collared owlet for breeding.

The first case of predation was on the evening of May 8 at 07: 21 p.m. In this case, a collared owlet entered a nest box (E46), killed and ate three nestlings (2 days old), and a brooding female green‐backed tit (Figure 1). The distance between the nest box of the owlet and this nest box (E46) was 112 m. At the time of this predation event, a female collared owlet was incubating its eggs; hence, the predating owlet might be the male from this pair.

On May 14, we initially found four nestlings of green‐backed tit in a nest box (G62). All four nestlings were raised safely until May 21; however, on May 22, 23, and 24, we found a decrease in the number of nestlings, losing one on each day. Although our cameras were set to capture 24‐h video recordings, the recording efficiency of the cameras decreased due to the rain, so we were unable to find any video of these events. However, on May 25, from video recording, we detected the case when a collared owlet preyed upon the fourth nestling from inside this box at 6: 51 (14–15 days old). This predated nest (G62) was located at a distance of 400 m from the collared owlet nest (E47). When this predation occurred, there was an owlet incubating eggs in E47, and it was not provided food afterward.

When we visited another nest of green‐backed tit (H06) on June 4 at 11:00 a.m., we found only three of the four nestlings. Using video analysis, we discovered a collared owlet preying on one of the four nestlings at 9:10 a.m. on June 4, while the adult female tit was not there (the nestlings were 11 days old). Since this nest box is located 4.4 km away from E47, we are unsure if it is the same owlet or a separate one. After the predation occurred in the third instance (H06), we reduced the hole size from 5 to 3 cm by adding a cover, to determine whether 3 cm is the appropriate size for green‐backed tit. The fact that the remaining three nestlings successfully fledged on June 13. This is indicative evidence that 3 cm works flawlessly.

In addition to the above three cases, nestlings from the other four nests of green‐backed tit were also predated. Unfortunately, the predators were unknown because no video tapings were available for those events. In all these cases, the artificial box's entrance hole was only 5 × 5 cm in size and had not been expanded or poked. However, we did not find any case of predation by collared owlet in the artificial nest boxes occupied by other cavity breeders.

## 4 DISCUSSION

In the present study, we found being the smallest owlet (15–17 cm), the collared owlet preyed on the breeding green‐backed tit and its nestlings in artificial nest boxes. In our study, we used artificial nest boxes with a 5.0‐cm entrance hole made it possible for the collared owlet to predate on adult brooding green‐backed tit and its nestlings (the first case). The entrance hole size that we used in our study is consistent with the research on the Japanese tit (*P. minor*) (Yin et al., 2023). Similarly, according to the Royal Society for the Protection of Birds (RSPB, https://www.rspb.org.uk), the hole diameter of the nest box for the Great tit (*P. major*) should be 2.8 and 2.5 cm for the coal tit (*Periparus ater*), marsh tit (*Poecile palustris*), and blue tit (*Cyanistes caeruleus*). The appropriate entrance size should be proportional to the body size of the target species because smaller entrances are crucially important to prevent predation events. Besides this, previous studies suggested there is a high danger for birds in cavity nests because there is only one route to escape when predators attack (Low et al., 2010). Even though tits have evolved a variety of nest defense behaviors to prevent their offspring and themselves from being harmed by predators, such as alarm calls, mobbing, and attacks (Yu et al., 2021), this tactic was not always effective enough to keep the predators away (Koosa & Tilgar, 2016; Møller et al., 2021; Wickler, 2013). The nest entrance is crucial for cavity‐nesting birds because it serves as a barrier against predators. If a hole's entrance is large enough, some predators, like chipmunks, snakes, and small owlets, can easily get into the nest cavity (Solheim, 1984; Suzuki & Ueda, 2013; Yu et al., 2020). Compared to a hole with a big entrance size, a cavity with a small entrance can deter more predators from entering and destroying the nest (Wesołowski, 2002). Therefore, we underline the significance of selecting a hole size appropriate (3 cm) for the body size of the green‐backed tit for its effective breeding in the future.

In conclusion, by presenting three cases of collared owlet depredation on brooding green‐backed tits and their nestlings in southwest China, our research provides a novel perspective on the early records of avian predators. Our observation highlights the need to reevaluate predator–prey interactions in tropical and subtropical regions in Asia.

## AUTHOR CONTRIBUTIONS

**Nehafta Bibi:** Investigation (lead); methodology (lead); writing – original draft (lead). **Qingmiao Yuan:** Methodology (supporting). **Caiping Chen:** Methodology (supporting). **Shaolian Chen:** Methodology (supporting). **Yubao Duan:** Investigation (lead); methodology (lead); writing – review and editing (supporting). **Xu Luo:** Funding acquisition (lead); investigation (lead); methodology (lead); project administration (lead); writing – review and editing (lead).

## 5 ACKNOWLEDGEMENTS

We are grateful for the logistic assist of the management bureau of Zixishan Nature Reserve in Chuxiong prefecture, Yunnan province.

## FUNDING INFORMATION

This study was supported by the National Natural Science Foundation of China (U23A20162) and the postdoctoral fund of Yunnan Province, China.

## CONFLICT OF INTEREST STATEMENT

The authors declare no conflict of interest.

## Data Availability

We will provided all required data files.
